# Biological resilience in health and disease

**DOI:** 10.1242/dmm.050799

**Published:** 2024-07-25

**Authors:** Helen Weavers

**Affiliations:** School of Biochemistry, Faculty of Life Sciences, Biomedical Sciences, University of Bristol, Bristol BS8 1TD, UK

**Keywords:** Stress adaptation, Hormesis, Cytoprotection, Cell survival, Degenerative disease, Healthy ageing, Antimicrobial resistance, Tumour adaptation

## Abstract

All living organisms – from single-celled prokaryotes through to invertebrates and humans – are frequently exposed to numerous challenges during their lifetime, which could damage their molecular and cellular contents and threaten their survival. Nevertheless, these diverse organisms are, on the whole, remarkably resilient to potential threats. Recent years have seen rapid advances in our mechanistic understanding of this emerging phenomenon of biological resilience, which enables cells, tissues and whole organisms to bounce back from challenges or stress. In this At a Glance article, I discuss current knowledge on the diverse molecular mechanisms driving biological resilience across scales, with particular focus on its dynamic and adaptive nature. I highlight emerging evidence that loss of biological resilience could underly numerous pathologies, including age-related frailty and degenerative disease. Finally, I present the multi-disciplinary experimental approaches that are helping to unravel the causal mechanisms of resilience and how this emerging knowledge could be harnessed therapeutically in the clinic.

## Teaser

**Summary:** This At a Glance article reviews how biological resilience mechanisms act across scales to protect organisms from harmful insults, focusing on the challenges and opportunities for harnessing this remarkable phenomenon therapeutically.

**Figure:**
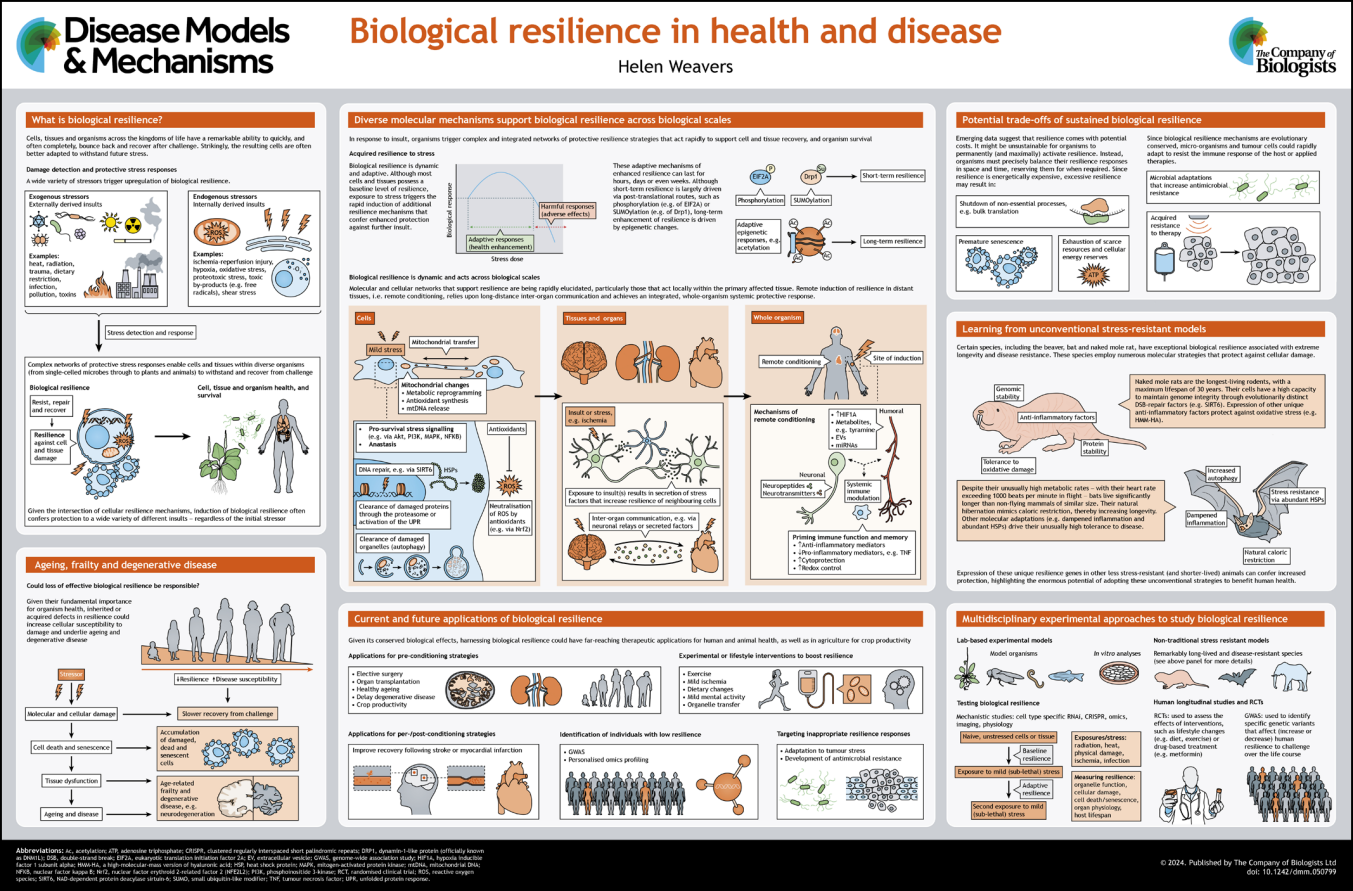
See Supplementary information for a high-resolution version of the poster.

## Introduction

During their lifetime, most cells and tissues in a variety of organisms – ranging from microbes to plants and animals – are frequently exposed to challenges that could cause damage, dysfunction and even compromise survival. These diverse challenges often originate from external sources, such as physical injury, infection, atmospheric pollution, temperature extremes or radiation, but can also arise internally as, e.g. free radicals from metabolism, errors during protein folding or local ischemia (see Box 1). Nevertheless, many cells and tissues show a remarkable resilience (see Box 1) to these insults, possessing the ability to quickly, and often completely, bounce back and recover once the stress has subsided (see poster, ‘What is biological resilience’) (Stone et al., 2018). Emerging evidence suggests this is because organisms have evolved complex, integrated networks of protective resilience strategies that act rapidly to support recovery and survival, so the organism can return to a state of homeostasis (see Box 1) (Holcombe and Weavers, 2022). These resilience mechanisms act across biological scales, from individual molecules through to cells, tissues and whole organisms (see poster, ‘Diverse molecular mechanisms support biological resilience across biological scales’). Strikingly, biological resilience is highly dynamic and adaptive. Although most cells and tissues possess a base-line level of robustness (see Box 1) and resilience, exposure to stress triggers rapid induction of additional resilience mechanisms that confer enhanced protection against further insult (Stone et al., 2018); this programmable or inducible nature of biological resilience makes it a particularly promising therapeutic entry point (Feder et al., 2019).

**Box 1. Glossary**

**Anastasis:** Phenomenon of cell recovery that rescues cells from the brink of death.

**Cellular health:** State in which cellular processes – e.g. mitochondrial activity, DNA/RNA/protein synthesis, membrane integrity, clearance of cellular waste etc. – proceed optimally.

**Cerebral ischemia:** Acute brain injury that results from impaired blood flow to the brain.

**Conditioning:** Form of cellular learning or memory in which a stimulus triggers adaptive responses that change (usually increase) the response to a second stimulus.

**Free radicals:** Highly reactive molecular species that contain an unpaired electron.

**Healthy ageing:** Process of developing and maintaining the functional ability that enables wellbeing (and disease resistance) in older age.

**Heat shock proteins (HSPs):** Large family of molecular chaperones with key roles in protein maturation, re-folding and degradation.

**Homeodynamics:** Dynamic form of homeostasis involving the constantly changing inter-relatedness of body components while an overall equilibrium is maintained.

**Homeostasis:** Self-regulating process by which a living organism can maintain internal stability while adjusting to changing external conditions.

**Hormesis:** Biphasic dose-response to an environmental agent, with low-dosage stimulation giving a beneficial effect and high-dosage stimulation giving an inhibitory or toxic effect.

**Hyperlipidaemia:** Excess of cholesterol or triglycerides in the blood.

**Ischemia:** Condition in which blood (and oxygen) flow is restricted or reduced in a part of the body.

**Ischemia-reperfusion injury:** Exacerbation of cellular dysfunction following the restoration of blood flow to previously ischemic tissues.

**Membrane strengthening:** Molecular mechanisms that increase the mechanical or chemical resistance of cellular membranes.

**Myocardial infarction:** Condition in which the supply of blood to the heart is suddenly blocked, (e.g. by a blood clot).

**Pentose phosphate pathway:** Metabolic pathway with roles in carbon homoeostasis, providing precursors for nucleotide and amino acid biosynthesis, and in the generation of reducing molecules, such as NADPH, to combat oxidative stress.

**Polygenic trait:** Characteristic or phenotype that is influenced by two or more genes.

**Polygenic resilience scores:** Profile of resilience-promoting genetic variants that protect against common diseases (e.g. Alzheimer's) within individuals with otherwise high genetic risk.

**Resilience:** Capacity of a biological system to recover after challenge, by either returning to the original state or establishing a new adapted state after perturbation.

**Robustness:** Ability of a biological system to resist deviation from its original state (e.g. resist detrimental effects) following challenge.

**ROS detoxification:** Process of detoxifying reactive oxygen species (ROS) by converting them to less-reactive products.

There has been a recent surge of interest in the mechanisms that govern biological resilience with the realisation of its enormous translational potential, particularly for curbing age-related disease and frailty (Travers et al., 2023). Human life expectancy has doubled in the past 100 years, meaning there are rapidly ageing populations with increased frailty, multi-morbidities and degenerative diseases (Garmany et al., 2021). A natural decline in the effectiveness of biological resilience with age may underlie many of these ageing phenotypes (López-Otín et al., 2023; Pyrkov et al., 2021). Moreover, plants and animals (including humans) are increasingly exposed to new, potentially damaging environmental threats, such as e-cigarettes and plastic pollution (Dick Vethaak and Legler, 2021; Lee et al., 2018). Thus, there is an urgent need to enhance resilience across the life-course to limit disease and prolong healthy ageing (see Box 1).

In this At a Glance article, I discuss current and emerging knowledge on the mechanistic basis of biological resilience, illustrating that resilience responses are conserved across the kingdoms of life, with certain species (such as the naked mole rat) possessing extraordinary resilience for remarkable longevity and disease resistance. I also present current and future experimental approaches to decipher the causal mechanisms of resilience, as well as its relationship to ageing and disease. Finally, I highlight the wide range of potential applications of resilience in the field and clinic, including improving post-operative recovery, treating degenerative disease, promoting healthy ageing, fighting anti-microbial resistance, and boosting crop productivity.

## Biological resilience is dynamic and acts across biological scales

Although most cells or tissues within organisms exhibit an intrinsic baseline level of resilience to stress, biological resilience is highly dynamic and adaptive (see poster, ‘Diverse molecular mechanisms support biological resilience across biological scales’). Indeed, after mild (sub-lethal) stress, organisms rarely return to their naive state; increasing evidence suggests that, upon initial detection of stress, cells rapidly induce numerous cytoprotective pathways that not only promote recovery from the insult directly at hand but drive longer-term adaptations that increase the resilience of the tissue or organism to further insult(s) (Holcombe and Weavers, 2022; Stone et al., 2018). This ability of organisms to respond, counteract and adapt to disturbance is often referred to as homeodynamics (see Box 1) (Yates, 1994), with the capacity (or ability) of a biological system to buffer these changes referred to as the homeodynamic space (Demirovic and Rattan, 2013). By stimulating compensatory and adaptive processes that render cells and tissues more resistant to subsequent hits, resilience enables organisms to quickly adapt to new challenges in their microenvironment (van der Weele and Jeffery, 2022). This stress adaptation is often referred to as conditioning or hormesis (see Box 1), as in Nietzsche's adage “What doesn't kill you, makes you stronger.” (Gems and Partridge, 2008; Schulz, 1888). Biological resilience pathways, therefore, collectively promote cellular health (see Box 1) and survival, limiting tissue dysfunction and disease progression.

Biological resilience protects from a multitude of different stressors that originate endogenously within the body, including oxidative stress, hypoxia, proteotoxic stress and free radicals, as well as from the environment, such as heat, radiation, trauma, altered nutrition, infection and pollution (see poster, ‘What is biological resilience?’). Strikingly, resilience responses occur across biological scales, with cytoprotective strategies acting both locally (within cells and tissues directly exposed to the stressor) as well as remotely in distant sites across the organism (see poster, ‘Diverse molecular mechanisms support biological resilience across biological scales’). Given that induction of biological resilience can confer protection to a wide variety of insults, it is not surprising that resilience involves complex intersecting signalling networks that affect multiple downstream cellular and physiological processes.

## Resilience at the molecular and cellular level

Successful induction of biological resilience relies on the ability of the cell to rapidly sense the insult (or its consequences) and then respond appropriately to the stressor for effective repair and recovery, with the effectiveness of these detection and response pathways determining the outcome, such as apoptosis, senescence, repair, growth or survival. These initial responses to insults begin with the molecular networks that detect stress and promote resilience, particularly those that act locally within the primary affected tissue(s) (see poster, ‘Diverse molecular mechanisms support biological resilience across biological scales’). Cells exposed to stressful insults upregulate numerous factors to help counteract and neutralise these threats, including antioxidants to neutralise free radicals, e.g. reactive oxygen species (ROS) or heat shock proteins (HSPs) (see Box 1) to prevent proteotoxic stress (Clemente and Weavers, 2023; Lang et al., 2021; Weavers et al., 2019). Free radicals (such as superoxide) are molecules containing unpaired electrons that are produced during normal physiology, e.g. as by-products of mitochondrial electron transport or as anti-microbial weapons by dedicated NADPH oxidase enzymes, but are greatly increased upon stress; since free radicals are highly reactive, left unchecked they can damage a multitude of cellular components (including DNA, proteins and lipids) (Clemente and Weavers, 2023; Holcombe and Weavers, 2023). Upon cellular stress, misfolded or unfolded proteins may also accumulate in the endoplasmic reticulum (ER), triggering clearance through the proteasome or activating the unfolded protein response (UPR); as part of the UPR, HSPs function as molecular chaperones, assisting in protein folding as well as protein degradation or protein disaggregation (Lang et al., 2021). In this way, the UPR and HSPs protect cells from damage to their proteome, i.e. from proteotoxic stress, that can occur following heat shock or exposure to proteotoxic compounds (e.g. heavy metals) (Lang et al., 2021). Upon cellular insult, numerous other protective mechanisms – including DNA-damage repair (e.g. base or nucleotide excision) (Kandel et al., 2024) and plasma membrane repair (Horn et al., 2017) – are triggered to quickly repair or remove damaged cellular components Depending on the extent of injury, damaged organelles, such as mitochondria, may either be repaired or cleared via cellular autophagy (Sokolova, 2018). Moreover, healthy organelles, may even be transferred from normal to stressed cells via specialised nanotubes to help restore specific cellular functions, as has been observed for mitochondria (Dong et al., 2023).

Cellular stress may also initiate metabolic reprogramming to reduce the production of toxic by-products, such as ROS, or increase production of metabolites that help neutralise ROS or contribute to cellular repair. For example, stressed renal cells minimise oxidative damage by diverting glucose into the pentose phosphate pathway (see Box 1) to increase availability of the cellular antioxidant glutathione and produce nucleotides for DNA synthesis (Holcombe and Weavers, 2023). Another major component of the protective response of the cell to oxidative stress involves activation of the transcription factor NFE2L2 (also known as Nrf2), a master regulator of the antioxidant response, which is normally targeted for degradation by its binding partner Keap1. Here, oxidative stress triggers ROS-dependent oxidation of cysteines within Keap1, relieving its inhibition of Nrf2 and enabling activation of downstream Nrf2-dependent stress responses (Schäfer and Werner, 2015).

Cellular stress may also trigger biomechanical changes, such as the strengthening or remodelling of plasma membranes, as observed in gram-negative bacteria that strengthen their outer membrane permeability barrier in response to stress and thus reduce antibiotic activity (Fernández et al., 2010; Llobet et al., 2011; Mitchell and Silhavy, 2019). Cells may also remodel their cytoskeleton, extracellular matrix (ECM) or nuclear envelope to make themselves more mechanically robust (van Haaften et al., 2020); for example, cells subjected to biomechanical stress can adapt their perinuclear cytoskeleton, nuclear envelope composition or chromatin structure to protect the genome against damage and promote cellular survival (Harada et al., 2014; Ju et al., 2023 preprint; Nava et al., 2020). Stressed cells may upregulate pro-survival (or anti-apoptotic) signalling, enabling cells to survive despite the presence of damage; for example, in response to challenge, neurons upregulate brain-derived neurotrophic factor (BDNF) signalling that promotes neuronal survival via the pro-survival protein Bcl-2 (Rothman and Mattson, 2013). Such pro-survival signalling may even enable cells to reverse apoptosis and recover from the brink of death, in a process known as anastasis (Sun et al., 2017). In this emerging phenomenon, remarkably, cells can survive caspase activation following sub-lethal apoptotic stimuli by upregulating a plethora of recovery pathways including TGFβ signalling and expression of the transcription factor Snail (SNAI1) (Sun et al., 2017).

These diverse adaptive mechanisms for enhanced resilience are controlled at transcriptional, translational, post-translational and epigenetic levels and, thus, can last for hours, days or even weeks (de Preux Charles et al., 2016). Since the detection of and response to stress must often be rapid, short-term resilience responses are largely driven by post-transcriptional and post-translational routes. Post-translational modifications (PTMs), such as phosphorylation and SUMOylation, are emerging as key factors enabling rapid stress detection and response (Ryu et al., 2020). By contrast, long-term enhancement of resilience is likely to be driven by epigenetic changes. Methylation of the HSP70 promoter, for example, has been linked to heat-stress-related epigenetic memory in organisms resilient to heat stress (Kisliouk et al., 2017), whilst the transcriptional repressor REST, a key regulator of the neuronal epigenome, promotes neuronal resilience to stress (see review by Mampay and Sheridan, 2019).

## Coordinating resilience across tissues

A striking feature of biological resilience is that local stress triggers remote induction of resilience in distant tissues, i.e. remote conditioning (see Box 1), thus achieving an integrated, whole-organism protective response. For example, non-lethal insults, such as ischemia-reperfusion injury (see Box 1) applied to specific tissues, e.g. through a restrictive cuff around a limb, can remotely condition distant organs – including the heart, kidney and brain – and protect them from further ischemic injury (Lim and Hausenloy, 2012). Although such systemic adaptations, undoubtedly, rely upon long-distance inter-organ communication, the underlying molecular mechanisms have remained more elusive. Traditionally, communication via hormonal and neuronal signalling pathways were considered the main routes driving remote conditioning. In this way, local stress can be communicated rapidly to distant sites by circulating chemical messengers, such as inflammatory mediators or metabolites (Cai et al., 2013; Chung et al., 2022), or through neuronal relays (Özbey et al., 2020). Emerging evidence now suggests that extracellular vesicles (EVs) provide an important conduit to trigger systemic resilience through delivery of protective miRNAs and proteins (Davidson et al., 2018; Minghua et al., 2018). Intriguingly, some evidence suggests enhanced resilience may even be passed across generations via epigenetic effects (Wan et al., 2021). For example, in the nematode *C. elegans*, heat shock not only increases the longevity of the treated organisms but increases the longevity of their descendants; such transgenerational inheritance relies on histone and DNA methyltransferases that prime stress response genes (e.g. heat shock factors) and promote their transcription to extend life span (Wan et al., 2021).

## Whole-organism resilience

The exact level of biological resilience within an organism also varies in space and time, over the course of the day, across the lifetime of or between different tissues within an organism. Recent work suggests biological resilience varies across the day with the circadian clock in animals (Early et al., 2018) and plants (Cano-Ramirez et al., 2023). Moreover, within an organism, certain cells and tissues that are most vulnerable to damage, such as metabolically active tissues with low intrinsic turnover like the kidney and heart (Wang et al., 2010), maintain higher levels of resilience (Burbridge et al., 2021); however, it remains unclear whether these tissue-specific differences in resilience are determined by organ identity or reflect dynamic adaptation to stress. Strikingly, certain species – including the beaver, bat and naked mole rat – have exceptional resilience, and this is associated with extreme longevity and disease resistance (Zhao et al., 2021; see poster, ‘Learning from unconventional stress-resistant models’). On a molecular level, these species employ numerous strategies that protect against cellular damage, including expression of evolutionarily distinct DNA-damage repair factors with increased activity, production of unique anti-inflammatory proteins (e.g. within the ECM) or increased expression of tumour suppressor genes (Takasugi et al., 2020; Tian et al., 2019; Zhang et al., 2021b) (see Box 2 for further details). Intriguingly, despite their large size and long lifespan, elephants are surprisingly resistant to cancer, a phenomenon known as Peto's paradox (Callier, 2019). This may in part be due to their increased copy number of the gene encoding the tumour suppressor TP53, which makes elephant cells extremely sensitive to detecting DNA damage and enabling efficient repair or elimination of damaged cells (Sulak et al., 2016). Bats, despite their high metabolic rates, also live significantly longer than non-flying mammals of similar body mass, which may be attributed to their natural hibernation habit, i.e. mimicking caloric restriction that increases longevity in other mammals (Wilkinson and South, 2002) and other molecular adaptations, such as dampened inflammation, that promote disease tolerance (Ahn et al., 2019). Thus, these organisms (particularly the naked mole rat and bat) are exceptional models for studying the cellular mechanisms underlying longevity and biological resilience (see Box 2).

**Box 2. The naked mole rat as a model for longevity and resilience**

Naked mole rats are known to be the longest-living rodents, with a maximum lifespan of 30 years, which is five times their predicted lifespan based on their body size alone (Edrey et al., 2011). Strikingly, this extreme longevity is accompanied by exceptional resistance to stress and disease (Zhao et al., 2021), making this unconventional species a powerful resource to uncover novel resilience mechanisms. Studies using this long-lived model are increasingly uncovering novel molecular mechanisms that can protect cells against cellular damage. Consistent with the notion that somatic DNA mutations are linked to ageing, single-cell whole-genome sequencing has revealed that naked mole rat cells have a higher capacity to maintain genome sequence integrity of both spontaneous and bleomycin-induced somatic mutations than shorter-lived species (Zhang et al., 2021a). In part, this might be due to their expression of evolutionarily distinct DNA-damage repair factors, such as the DNA double-strand break (DSB)-repair factor SIRT6 (Tian et al., 2019). Naked mole rats also produce unique high-molecular-mass versions of major ECM components, such as a high-molecular-mass version of hyaluronic acid (HMM-HA), that are anti-inflammatory and protect against oxidative stress (Zhao et al., 2023b). Together with increased expression of longevity-associated genes and tumour suppressor genes within aged tissues (Fatima et al., 2022), this is probably why naked mole rats are particularly disease-resistant. Promisingly, expression of naked mole rat resilience genes in rodents with shorter lifespans can confer increased stress resistance. For example, overexpression of the naked mole rat version of hyaluronan synthase 2 (Has2) promotes increased cancer resistance and longevity in mice (Zhang et al., 2023). Such studies highlight the tremendous potential of adopting these unconventional strategies to benefit human health.

## Could loss of biological resilience underlie ageing, frailty and degenerative disease?

Given the fundamental role of biological resilience in sustaining cellular health within animals and plants, acquired or inherited defects in resilience mechanisms could increase cellular susceptibility to damage – or senescence and death – and underlie numerous diseases (see poster, ‘Ageing, frailty and degenerative disease’). Although ageing has traditionally been considered a consequence of inherently imperfect cellular repair mechanisms that lead to a time-dependent accumulation of damage (López-Otín et al., 2023), emerging evidence now suggests that age is associated with declining biological resilience; indeed, following the same challenge, older individuals are well-known to recover less efficiently compared to their younger counterparts (Berian et al., 2016; Ukraintseva et al., 2021). Ageing could, thus, be considered a consequence of the shrinkage of the so-called homeodynamic space (Demirovic and Rattan, 2013). Such loss of biological resilience and progressive accumulation of cellular damage is likely to be tightly linked to the evolution of the essential lifespan of a species, i.e. the average limited lifespan of a species under natural conditions (Rattan, 2024).

Older individuals are now thought to have a reduced ability to sense as well as respond to challenges that normally trigger robust responses of resilience (see Box 1), and this could be a key factor driving age-related frailty, multimorbidity and degenerative disease, such as neurodegeneration (Ukraintseva et al., 2021). For example, cellular antioxidant machinery has reduced effectiveness against oxidative stress in aged cells and tissues (Meng et al., 2017). Compared to their younger counterparts, aged cells also display marked metabolic inflexibility that predisposes them to cellular stress and damage (López-Otín et al., 2023). Currently available tools to predict individual resilience rely on diagnostics that aim to establish biological age (Levine, 2013), by analysing epigenetic clocks (Hannum et al., 2013) or plasma biomarkers (Pyrkov et al., 2021). However, the decline in biological resilience in older individuals is exceptionally heterogeneous, making it difficult to predict which individual could benefit the most from enhanced resilience. Nevertheless, the rising number of centenarians and supercentenarians (people over 110 years) demonstrates that it is possible for humans to live well for a long time; these individuals possess particularly robust resilience and have managed to recover from the challenges they have experienced over their lifetime (Lin et al., 2021; Simon et al., 2022). In fact, recent omics profiling of immune systems across the life-course suggests centenarians are particularly successful at adapting to stressful insults and this may underlie their exceptional longevity (Karagiannis et al., 2023). If we can uncover why these individuals are so resilient at molecular level, these molecules could be powerful therapeutic targets (see poster, ‘Current and future applications of biological resilience’).

## Current and future applications of biological resilience

Given its widespread conserved biological effects, harnessing biological resilience could have far-reaching therapeutic applications for human and animal health, as well as in agriculture for crop productivity (see poster, ‘Current and future applications of biological resilience’). The ‘cross-inducible’ nature of biological resilience – with one stressor inducing protective responses that confer resilience to a wide variety of other stressors, together with its long-range, i.e. remote, effects – makes it a particularly promising therapeutic entry point for augmenting stress resistance in the clinic. Interventions to boost resilience might be implemented before, during or after the insult (also referred to as pre-, per- or post-conditioning, respectively), depending on the (un)predictability of the stressor. In the clinic, boosting biological resilience before elective surgical procedures could help accelerate patient recovery or improve the success of organ transplantation (Veighey et al., 2019). Augmented resilience could also enhance recovery following unplanned adverse health events, such as cerebral ischemia or myocardial infarction (see Box 1) (Zhao et al., 2023a), or delay the onset of degenerative disease or neurodegenerative conditions, such as Alzheimer's disease (Selvaraji et al., 2019). Given our ageing population, there is now considerable focus on promoting healthy resilient ageing. With molecular insight, could we boost human biological resilience to mimic that of certain long-lived disease-resistant species? Recent evidence is already suggesting such strategies can succeed. Akin to the effects of the unique *Sirt6* gene found in long-lived naked mole rats (see Box 2) (Tian et al., 2019), a population of centenarians was found to be overrepresenting a specific allele of the *SIRT6* gene containing two missense mutations, which enhances repair of DNA double-strand breaks (DSBs) and the killing of cancer cells (Simon et al., 2022). Age-related frailty may also be halted – or even reversed – by interventions designed to boost resilience, such as mild physical exercise, sub-lethal bouts of ischemia, nutritional changes (e.g. dietary restriction) or mild mental activity (Travers et al., 2023).

As our mechanistic knowledge of the molecular basis of resilience grows, pharmacological interventions are also being trialled as more-targeted strategies to boost cellular resistance to and recovery from stress. These strategies include administration of the FDA-approved drug metformin, which mimics the effects of dietary restriction and prolongs lifespan in animal models by activating numerous stress responses (Barzilai et al., 2016), as well as rapamycin, a potent inhibitor of the mTOR protein kinase that regulates cellular metabolism, growth and survival (Mannick et al., 2018). More recently, novel small-molecule drugs (known as senolytics) that selectively clear senescent cells are being trialled to ameliorate a range of age-related diseases (Justice et al., 2019; Kirkland and Tchkonia, 2020). A more radical approach has now emerged with the transplantation of fully functional organelles, particularly mitochondria, into patient cells (McCully et al., 2016). By transferring isolated healthy mitochondria, this strategy aims to rescue the health of defective cells – such as neurons of individuals with common neurodegenerative conditions – that demonstrate mitochondrial dysfunction (Lightowlers et al., 2020). Remarkably, such an approach is showing potential promise using intravenous introduction of healthy mitochondria to treat individuals with myocardial ischemia-reperfusion injury, spinal cord injury and the neurodegenerative disorder Parkinson's disease (Lightowlers et al., 2020). However, for effective application of resilience in the clinic, it will be important to determine the appropriate level, i.e. intensity or duration, to which these protective pathways should be activated. Indeed, emerging evidence suggests that prolonged or excessive activation of resilience pathways can have detrimental impacts on tissue health (see Box 3).

**Box 3. Trade-offs associated with elevated biological resilience**

Since biological resilience sustains cell health in the face of a multitude of insults, why are cells within an organism not permanently maintained in a state of maximal resilience? Resilience could come with potential costs; thus, it may be unsustainable for organisms to permanently activate resilience. Instead, organisms must precisely balance their resilience responses in space and time.

**Energetically expensive and exhaust scarce resources**

Cellular stress responses are energetically demanding. In times of stress, synthesis of stress-response proteins is prioritised over non-essential processes, leading to a general shutdown of translation (Williams and Rousseau, 2024). Thus, experimental approaches that constitutively elevate resilience can cause long-term detrimental effects on cell health, including the induction of senescence (Hiebert et al., 2018). Although dietary restriction (DR) is well-known to induce conserved pro-longevity responses, recent evidence suggests DR-driven adaptations have hidden costs that are revealed when the stressor is removed (McCracken et al., 2020). Such trade-offs could contribute to the male–female health-survival paradox, i.e. better health but worse survival in men compared with women. Males may be more resilient in middle life but have lower survival rates in old age because they invest more reserves for resilience in their youth (Oksuzyan et al., 2008).

**Acceleration of tumour growth**

Although the repair of molecular damage is tumour suppressive, pro-survival signalling can enable survival of cells that should otherwise be removed. Tumour cells can also activate resilience pathways following exposure to stressful insults (e.g. radiotherapy), which could enhance tumour growth and promote resistance to radio- or chemotherapy (Wang et al., 2023).

**Antimicrobial resistance**

Biological resilience mechanisms are evolutionarily conserved. Thus, microorganisms can rapidly adapt to the microbiocidal armoury of the host, exacerbating antimicrobial resistance. Bacterial species, such as *Staphylococcus aureus*, can adapt to resist oxidative damage from the ROS-mediated immune attack of the host (Podkowik et al., 2023), while gram-negative bacteria are able to strengthen their outer-membrane permeability barrier during stress, a main barrier to antibiotic activity (Mitchell and Silhavy, 2019).

A major ongoing challenge is to understand how confounding factors, e.g. co-morbidities, co-medications or age, impact the response of an individual to resilience-inducing interventions. It has become clear that certain risk factors, including chronic inflammation, diabetes and hyperlipidaemia (see Box 1), reduce the effectiveness of pre-conditioning, probably explaining the disappointing outcomes of large clinical trials testing cardioprotective interventions (Ferdinandy et al., 2023; Schulz et al., 2020). Although certain pharmacological agents, e.g. metformin, have pro-longevity effects when applied early in life, recent work suggests these same agents cause life-shortening toxicity when provided in late life, as they exacerbate ageing-related mitochondrial dysfunction (Espada et al., 2020). This highlights the clear need to test novel resilience-boosting interventions in clinically relevant models of various risk factors, i.e. co-morbidities and co-medications, so that interventions can be better targeted for specific patient populations. Finally, as well as designing interventions that boost resilience, researchers are beginning to use their knowledge to selectively weaken the resilience of specific cell types. For example, increasing evidence suggests that cancer cells hijack resilience strategies to evade immune or therapeutic attack (see Box 3) (Wang et al., 2023). Thus, selective inhibition of resilience pathways within tumours could offer a powerful strategy to overcome tumour-acquired resistance. A similar approach could help combat antimicrobial resistance by specifically blocking microbial resilience strategies, such as ROS detoxification or membrane strengthening (see Box 1) (Mitchell and Silhavy, 2019; Podkowik et al., 2023).

## Multi-disciplinary approaches to study biological resilience

A full mechanistic understanding of the integrated molecular, cellular and physiological networks that underlie biological resilience requires the integration of multiple disciplines (see poster, ‘Multidisciplinary experimental approaches to study biological resilience’). Cell-based *in vitro* studies have proven invaluable for understanding the molecular networks that support cellular repair and recovery following insult, particularly due to the plethora of stressors that can be investigated, such as heat, ROS, radiation, hypoxia or mechanical injury. The effect of such stressors can then be investigated using a range of genetically modified cell types and rapidly assessed using well-characterised stress response assays. Such studies have begun to reveal the molecular pathways that promote cell recovery from cell death, i.e. anastasis (Sun et al., 2017), as well as numerous protective mechanisms affecting cellular metabolism, organelle clearance or transfer, DNA and plasma membrane repair, and limitation of proteotoxic or oxidative stress (Dong et al., 2023; Horn et al., 2017; Lang et al., 2021; Sokolova, 2018; Verma et al., 2021). As well as characterising intrinsic cellular stress responses, *in-vitro* cell culture is also helping to reveal the mechanisms by which stressed cells may coordinate organismal-wide responses through release of extracellular signals such as exosomes (Davidson et al., 2018).

Moving to *in-vivo* studies, preclinical studies of physiological resilience networks, particularly those underlying remote conditioning, have traditionally utilised larger mammalian models (Hausenloy et al., 2016). By contrast, experimentally tractable and comparatively simple *in vivo* models, such as zebrafish, *C. elegans* and *Drosophila*, are now growing as powerful tools to dissect how resilience responses act across scales (Bise et al., 2019; de Preux Charles et al., 2016; Holcombe and Weavers, 2023; Özbey et al., 2020). Although these simpler experimental models do not display the genomic and cellular complexity of humans, the comparative ease of rapid, large-scale non-invasive screening approaches offers unrivalled opportunities to identify novel resilience candidates. For example, recent live-imaging and genetic studies in *Drosophila* revealed the signalling pathways that boost Nrf2-mediated resilience within damaged epithelia to protect against collateral oxidative damage from the accompanying inflammatory response (Weavers et al., 2019). Similar Nrf2-driven antioxidant defences function in *Drosophila* immune cells to curb premature immune senescence (Clemente and Weavers, 2023). Zebrafish are also proving a valuable tool to uncover the signalling networks that support remote cardioprotection (Bise et al., 2019; de Preux Charles et al., 2016), whilst work in *C. elegans* has helped reveal how stress-inducible transcription factors, e.g. XBP1, promote systemic stress responses via extracellular release of key signalling factors, such as the amino acid tyramine (Özbey et al., 2020).

Despite significant advances in our mechanistic understanding of biological resilience in recent years, further research is still required before the therapeutic potential of resilience can be completely realised. Going forward, it will be crucial to identify the individuals most vulnerable to ageing or disease due to their lack of resilience. Indeed, the potential costs associated with sustained, excessive biological resilience (discussed in Box 3) mean we must identify those individuals who would benefit most from enhanced resilience, such as those genetically predisposed or with occupational hazard exposure. Genetic epidemiology approaches are already showing promise in identifying genetic variants – i.e. by using polygenic resilience scores (see Box 1) – that affect the resilience of an individual to developing degenerative conditions (Hou et al., 2022). To measure the resilience of a person, new methods that consider its dynamic and adaptive nature are also required. Rather than assessing steady-state parameters, we must measure the ability of an individual to respond dynamically to stress. Given that biological resilience acts across scales and has powerful long-range systemic effects, we must also fully understand resilience at a ‘systems level’. Can we identify strategies that not only boost the resilience of individual cells or tissues but that also impact the resilience of the whole organism?

Lately, research utilising unconventional stress-resistant and exceptionally long-lived animal models (such as the naked mole rat) have revealed novel factors that enhance resilience, such as those involved in DNA-damage repair, detoxification and metabolism (Zhao et al., 2021) (see also Box 2). Strikingly, rare human alleles of pro-longevity genes, such as that of the DNA-damage repair factor SIRT6, are now linked to elevated cellular stress resistance in centenarians (Simon et al., 2022). The recent revolution in human omics (particularly genetics) studies, with ever-growing numbers of genome-wide association study (GWAS) cohorts and human biobanks, offers new prospects to identify resilience pathways with major relevance for humans. Here, longitudinal monitoring of human populations will be important to capture how and why resilience changes dynamically across the life course (Karagiannis et al., 2023). Nevertheless, the complexity of cellular resilience responses means that the overall risk of an individual is a highly polygenic trait (see Box 1), a fine balance of resilience-promoting and resilience-impeding genetic variants (Hou et al., 2022). Such complexity, combined with the ever-increasing volume of omics data being generated, is necessitating more sophisticated bioinformatic approaches, such as deep learning-based tools, for data analysis (Sayed et al., 2021). These *in silico* approaches must be combined with randomised clinical trials (RCTs) to measure the effectiveness of new interventions on specific populations. For example, a recent RCT has shown primary care interventions, such as exercise or increased dietary protein, for older adults can build resilience and reverse frailty (Travers et al., 2023). Collectively, we envision that these multi-disciplinary approaches will enable the identification of prognostic markers of resilience, as well as the development and testing of new clinical interventions to boost resilience.

## Conclusions

Biological resilience is a complex and dynamic phenomenon that enables cells, tissues and whole organisms to recover from stressful insults. At the molecular level, it involves the coordinated interaction of numerous signalling pathways that enable cellular components to resist and repair damage, collectively promoting cell survival. At the systemic level, these local and acute stress responses are communicated more widely across the organism to promote long-term adaptation to future stress. These protective responses are key to sustained health. However, loss of biological resilience is increasingly linked to the development of age-related degenerative conditions and the frailty of older individuals. Although, in recent decades, multi-disciplinary approaches have enabled this field to make considerable progress, clinical translation of resilience remains a key unmet challenge despite its enormous therapeutic potential. In the near future, we envision that, more focused, personalised approaches that enable researchers to decipher the most appropriate interventions to boost resilience in specific populations will substantially advance the current perspectives for clinical implementation.

## Poster

Poster
