# Supplementary figures and images for: Biological resilience in health and disease
Source: Dis Model Mech. 2024 Jul 25;17(7):dmm050799. doi: 10.1242/dmm.050799 (PMC11552498; doi:10.1242/dmm.050799)

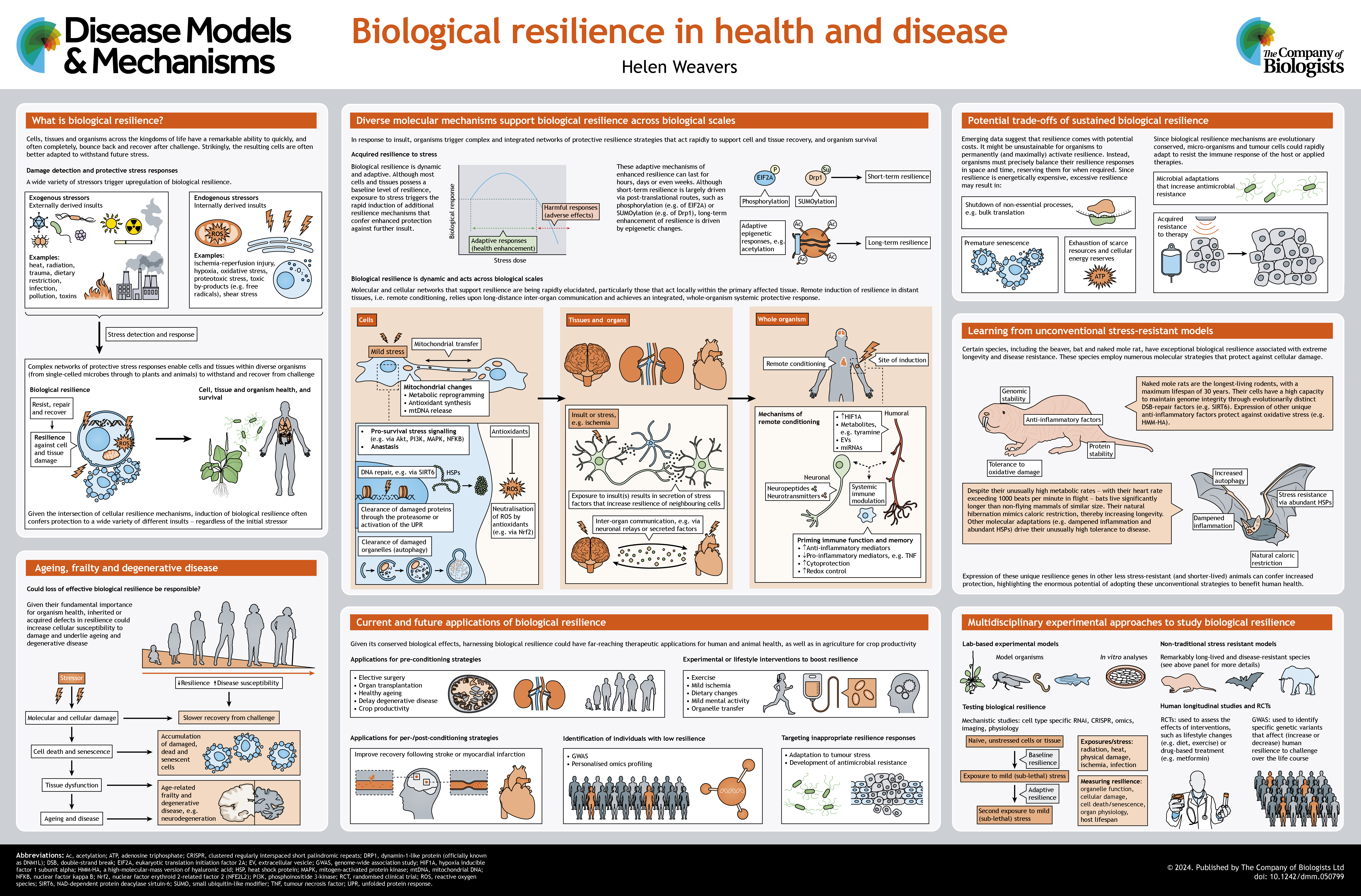

Supplement: Poster [file dmm-17-050799-s1.jpg]
